# Paediatric Critical Care Implementation in Lower-Resource Settings: Application of Community-Based Group Model Building

**DOI:** 10.21203/rs.3.rs-10133341/v1

**Published:** 2026-09-10

**Authors:** Yudy Fonseca, Terrie Taylor, Josephine Langton, Emma Thomson, Tiyamike Kapalamula, Adrian Holloway, Adnan Bhutta, Fiona Muttalib, Nicole O'Brien, Douglas Postels, Allan Doctor, Ellis Ballard, Kenneth E. Remy

**Affiliations:** 1. Division of Critical Care, Department of Pediatrics, University of Maryland School of Medicine, Baltimore, MD, USA; 2. Department of Osteopathic Medical Specialties, College of Osteopathic Medicine, Michigan State University, East Lansing, MI, USA; 3. Department of Pediatrics, Queen Elizabeth Central Hospital, Blantyre, Malawi; 4. Department of Pediatric Surgery, Queen Elizabeth Central Hospital, Blantyre, Malawi; 5. Department of Pediatrics, Indiana University School of Medicine and Riley Children's Health, Indianapolis, IN, USA; 6. Division of Pediatric Critical Care Medicine, Department of Pediatrics, BC Children's Hospital, Vancouver, BC, Canada; 7. Division of Critical Care Medicine, Department of Pediatrics, Nationwide Children's Hospital, The Ohio State University, Columbus, OH, USA; 8. Division of Neurology, George Washington University/Children's National Hospital; Washington DC, USA; 9. Brown School of Social Work and Director of the Social System Design Lab, Washington University, St. Louis, MO, USA; 10. Department of Pediatrics, Rainbow Babies and Children's Hospital, Cleveland, OH, USA; 11. Department of Internal Medicine, University Hospitals of Cleveland, Cleveland, OH, USA

**Keywords:** Global Health, Group Modeling, Systems dynamics, PICU, LMIC

## Abstract

**Objectives:**

Given the significant burden of critical illness and high mortality in Low-Middle Income Countries (LMICs), ICU care could substantially improve patient outcomes in LMICs. No systematic approach is available to identify the perceived and actual challenges of developing and maintaining high-quality intensive care units in LMICs. This study aimed to use community-based group model building (GMB) as an intervention to evaluate the barriers and facilitators of establishing a Paediatric Intensive Care Unit (PICU) in LMIC.

**Design:**

A GMB workshop was conducted with 27 participants, representing all the stakeholders. The workshop objectives were: assess GMB as a tool for implementation planning in a lower resource clinical care setting; assess barriers and facilitators of PICU implementation as perceived by conference attendees; develop a shared vision for goals, processes, and challenges for the PICU.

**Setting:**

PICU at the Mercy James Center for Paediatric Surgery and Critical Care (MJC) within the established system of Queen Elizabeth Central Hospital (QECH) in Blantyre, Malawi

**Interventions:**

None

**Main Results:**

The GMB workshop produced a synthesized causal map comprised of the individual participatory groups' causal maps. Participants created actionable ideas to promote collaboration or mitigate deleterious feedback loops undermining productivity.

**Conclusions:**

Group model building is valuable as a participatory process involving multiple stakeholders in understanding challenges in implementing, functioning, and sustainability of a PICU in lower resource settings. The flexibility in designing the GMB workshops to incorporate the voices of different stakeholders and openly address their concerns made it a valuable participatory process in addressing various perceived challenges in implementing and sustaining high-quality PCCM in lower resource settings.

## Introduction

The speciality of critical care in adults began in high-income countries (HICs) during the polio epidemic at the beginning of the twentieth century when the need for dedicated monitoring and continuous care exceeded hospital floor practices (1-4). Critical care for children was established by the 1950s (1, 2). Paediatric critical care in low- and middle-income countries (LMIC) began with the opening of the first African intensive care unit (ICU) in 1969 (1, 5). However, as HIC ICUs have advanced, ICUs in LMIC have not followed the same trajectory (3, 5). A 2014 systematic review found that the number of paediatric and adult ICU beds, as a percentage of hospital beds, was about 1.5% in LMIC compared to 9% in HIC (6). This relative paucity of ICU beds is in contrast with LMIC's higher burden of critical illness (1, 3-5, 7). In 2017, 73% of the 5.4 million deaths among children under five occurred in LMICs (8). Children with critical illnesses benefit from basic ICU care, such as a higher nurse-to-patient ratio, effective shock management, and closer patient monitoring (3, 5, 9-11). However, substantial barriers exist to providing high-quality intensive care units in LMICs (1, 2, 5, 10).

Previously published work used a contextual framework to identify perceived barriers to ICU care in LMICs:1. Properly trained and compensated *staff;* 2. Appropriate medical *equipment*; 3. Adequate sterilization and clean *spaces* to treat patients; and 4. *Systems* capable of providing higher levels of servicer (1). Lack of staff is a problem in both HICs and LMICs, with an estimated lack of 10.3 million health workers worldwide; however, this challenge is more apparent in rural areas of LMIC, where 70% of the deficit occurs (5, 10, 11). In LMICs, training in critical care is limited or non-existent, requiring staff to travel to higher-resourced environments, creating an opportunity for staff emigration (1, 10). Procuring appropriate equipment and maintaining clean spaces is a challenge in LMICs due to the need to establish ICUs within overextended healthcare systems that face high demand in a substandard infrastructure (1, 5, 12). However, ICUs need not be technologically advanced to impact outcomes positively. Given the high burden of critical illness and mortality in LMICs, basic ICU care could significantly improve patient outcomes(1, 2, 5, 10, 13).

Our team collaborated on the design, training, and initial implementation phases of the first PICU in Blantyre, Malawi, located at the Mercy James Center for Paediatric Surgery and Critical Care (MJC) within Queen Elizabeth Central Hospital (QECH). To assess the challenges and opportunities involved in establishing pediatric ICUs in LMICs, we utilized system dynamics, a method tailored to understanding the behaviour of complex, dynamic systems. Community-based group model building (GMB), a participatory approach within system dynamics, was employed to engage diverse stakeholders in analyzing the system's intricacies. Through this process, we sought deeper insights into the barriers and feasibility of implementing a PICU in this low-resource environment. (14, 15)

## Methods:

QECH is a tertiary referral hospital in southern Malawi with 13,000 annual admissions. Malawi is one of the world's poorest countries, consistently falling in the top 10 of impoverished nations (16). MJC was developed and opened on July 11, 2017, to address acute care and critical illness in children in Malawi. MJC has Malawi's only stand-alone PICU, a six-bed, technologically advanced unit with bedside monitors, ventilators, and oxygen. The nursing staff consisted of nurses with paediatric experience and recent nursing school graduates, all of whom received critical care training. Physician coverage included an anesthesiologist from the Paediatric Anesthesia Department at QECH and rotating paediatric intensivists from Norway through the Blantyre Oslo Project. Other stakeholders involved in the PICU establishment and functioning included the Paediatric Surgical Department, Paediatric Anesthesia Department, General Paediatric Department, Kamuzu College of Nursing, Blantyre Malaria Research Project (a research affiliate within the University of Malawi College of Medicine), and three non-governmental organizations (NGOs) Raising Malawi, Physicians for Peace, and Blantyre Oslo Project.

In October 2017, three months after the PICU opening, we conducted a GMB workshop with 27 participants, including representatives from all the stakeholders. Two facilitators trained in GMB at the Social System Design Laboratory at the Brown School at Washington University in St.Louis (USA) led the sessions. The Washington University institutional review board reviewed this project and waived the need for approval and written consent to participate. All participants provided verbal consent to participate during the group model-building sessions. There were no participants under the age of 16 years. Participation was voluntary and could withdrawn at any time.

Workshop objectives were:

1. Assess group model building as a tool for implementation planning in a lower resource clinical care setting.
2. Assess barriers and facilitators of PICU implementation as perceived by conference attendees.
3. Develop a shared vision/ model/ language for goals, processes, and challenges for the PICU.

Two separate GMB sessions were conducted over the two days, each with different stakeholder groups. The focus of the sessions was to conceptualize the underlying problem structure of opening and operating a high-quality paediatric ICU through a set of structured GMB *scripts*. Scripts are structured frameworks designed to facilitate group discussions by outlining steps for organizing conversations and collecting and analyzing data (17, 18).

Sessions began with an orientation to system dynamics and a review of GMB's fundamental principles and language. Next, the mediators elicited perceived facilitators and barriers to ICU establishment from attendees and assessed how these vary through time. These were shared, and participants worked together to create a consensus list of facilitators and barriers most influential in creating a high-quality paediatric ICU. Finally, participants were divided into teams, and individual *causal loop diagrams* were created using the facilitators and barriers consensus list. The facilitation team synthesized the individual causal maps into one preliminary causal loop diagram (Figure 4). A *causal loop diagram* is a qualitative model visually representing the interactions between facilitators and barriers, illustrating causal relationships and highlighting reinforcing or balancing feedback loops within a system (15). In causal loop diagrams, arrows represent causal links between system variables. The small '+' or '−' beside the causal links describes its polarity. Examples:

- The link between available staff and the staffing gap has a **negative causal link (purple arrows with - sign)**, meaning that the staffing gap decreases as available staff increases. The inverse is also true: as available staff decreases, the staffing gap increases (Figure 1).
- The link between patients coming through the door and the staffing gap has a **positive causal link (purple arrows with + sign)**, meaning that the staffing gap increases as patients coming through the door increase. Again, the inverse is true (Figure 1).
- A **reinforcing loop** is when an action produces a result that influences more of the same action. For example, launching new initiatives increases the target for patient outcomes, increasing the gap in patient outcomes and bringing in new research initiatives (Figure 2).
- A **balancing loop** occurs when an action on a variable causes changes that eventually counteract the initial action. For example, as the number of admitted patients rises, staff workload increases, leading to more errors and lower quality of care. This decline in care reduces the hospital's reputation, causing fewer patients to seek admission. The equal sign in loop diagrams indicates a delay, such as the delay between patient outcomes and changes in reputation in Malawi. (Figure 3).

The next day, participants discussed and revised the initial causal loop diagram to identify facilitators and barriers and develop a shared mental model. After the model critique, the workshop closed with participants developing actionable ideas to intervene in the causal loop diagram. The group plotted the interventions on axes from *Low Impact* to *High Impact* and *Easy to Do* to *Hard to Do*.

## Results:

The GMB workshop produced a synthesized causal map comprised of the individual participatory groups' causal maps. (Figure 4 & Table 1)

Workshop output also included **actionable ideas** to promote collaboration or mitigate deleterious feedback loops (*Table S1 Actionable Ideas*). Actionable items were categorized into three groups, accompanied by an assessment of their ease of implementation. The *first category* assessed how to manage external influences' positive and negative effects on departmental and hospital levels. Simple suggestions included on-the-job training with dedicated educators and creating spaces for staff to discuss patients at the bedside and in meetings. The group also explored the importance of formal and informal discussions for raising cultural awareness. More challenging actions involved developing a standardized curriculum for critical care training, certification, and a continuous quality improvement program, which includes patient safety initiatives like good catch and stop-the-line programs to help staff address potential safety issues. The *second category* focused on reducing the negative impacts of establishing a high-resource centre within a low-resource hospital. It emphasized improving the partnership and communication between the PICU and QECH. Easy steps included sharing educational opportunities, activities, and resources. More complex tasks involved rotating staff between units, setting up a high-dependency unit for the pediatric department, and creating and staffing a resuscitation room. The *third category* examined how to lessen the impact of the new PICU on local staff at departmental and hospital-wide levels. Key actions included building and strengthening relationships among staff by creating opportunities for peer education.

## Discussion:

There has been no systematic approach to identifying the perceived and actual challenges of developing and maintaining high-quality intensive care units in LMICs. While the challenges to *staff*, *equipment*, *space* and *systems* exist to varying degrees at each institution, each is a unique system with complex dynamics. In this evaluation, we used Community-based GMB at QECH to help stakeholders create a shared model for improving and maintaining a high-quality PICU. GMB was employed to understand challenges in PICU development and to identify facilitators and barriers to implementing GMB in a low-resource setting. Multiple key process insights emerged from the participatory workshop and the causal map.

Participants had varying views on what constitutes positive patient outcomes. Some associated outcomes with the total number of admissions, while others focused on factors like disease severity and mortality risk. There was also a discussion about including post-hospital morbidity in the definition. These differences highlighted the need to align stakeholders' definitions of success. The workshop facilitated a discussion that led to a collectively agreed-upon definition of success and the creation of a shared model.

Additionally, participants discussed the benefits and challenges of new initiatives and the inclusion of non-Malawian personnel in research and clinical care teams. Historically, QECH has attracted non-Malawian personnel and new medical research initiatives. The benefits of these initiatives were seen in increased human capital, financial resources, and more advanced medical procedures and equipment, all of which were perceived as improving patient outcomes. However, the rapid implementation of new medical initiatives was thought to exacerbate the existing capability and knowledge gap among staff, leading to negative consequences for staff and patient care. This accelerated pace could also strain research teams, creating conditions of limited capability and resources and hindering successful implementation. Moreover, the presence of non-Malawian personnel in leadership roles can contribute to cultural and linguistic gaps and a lack of long-term consistency in PICU leadership. These challenges can lead to issues in staff coordination within and across departments, ultimately affecting the quality of patient care. To address this, non-Malawians in leadership positions must recognize and value the expertise and experience of Malawian staff. Gaining 'buy-in' from the local team is essential for any intervention's long-term success and sustainability (1).

Attendees were concerned about the high cost of resources needed to develop and maintain critical care services. The concern was that already limited resources, especially funding and nursing, would be perceived as being drawn away from the lower-resourced hospital to the high-resourced PICU, creating a resource gap between settings. A perceived resource gap could increase frustration and negatively impact the quality of communication and cooperation between departments. To mitigate this, leadership needs to address the limited resources and their allocations when building a high-resource department within a low-resource hospital.

Historically, training has been considered the key solution to enhance staff capabilities and knowledge, aiming to narrow the capability gap. However, a critical insight from the workshop was that while crucial, traditional training methods like didactics alone are inadequate in bridging this capability gap. Training approaches from HICs do not usually consider the risk of high staff turnover and the lack of a coordinated system that could provide opportunities for ongoing peer learning. When identifying intervention points that will improve system function and elevate staff knowledge and capability, it is necessary to consider multiple mechanisms for capacity development aside from training, such as staff retention (7, 10).

Our study shows that GMB is feasible in low-resource settings. The structure of GMB allowed groups, usually not empowered to speak, to provide essential input. Understanding the hospital system's complexity was achieved by having a wide breadth of workshop participants and allowing attendees at different clinical care and supervisory levels to partake in a constructive dialogue. Another critical insight into the process was the value of boundary objects like the causal map in negotiating disparate mental models between stakeholder groups. The causal map exposed the underlying assumptions and perspectives about the problems different stakeholders were facing. Additionally, the boundary objects allowed new stakeholders to understand better the system in which they would be working and the dynamics between the in-country teams. Lastly, the role of an independent mediator was crucial to maintaining sufficient neutrality in listening to various stakeholder groups' voices and ensuring that the process was open and transparent to all stakeholders. A mediator created a space that allowed all stakeholders, especially those with less power, to speak up and give their opinions.

The limitations of the projects were the ability of mediators to create enough knowledge on the concept of group modelling and system dynamics for an audience that had no prior exposure and whose interest was unclear. Additionally, limitations to the success of the GMB process included the absence of local input during the project's initial inception, the lack of in-country champions, and the timing of the project. Given the lack of local input, the initial focus of the workshops centred on the research team's goals; however, upon the facilitator's arrival in Malawi, the objective was adjusted to prioritize attaining objectives set by multiple stakeholder groups with an emphasis on local team engagement. The absence of a local champion impeded meaningful dissemination within the country, potentially diminishing the impact of the study's findings. Lastly, the GMB's timing prevented the PICU's future leadership from being present to aid in creating the shared model.

## Conclusions:

In this prospective qualitative study, we demonstrate the ability and benefit of the GMB process to support the implementation and sustainability of high-quality pediatric critical care medicine (PCCM) in lower resource settings. GMB allowed the evaluation of perceived barriers to identify them in MJC and created a therapeutic platform for hospital staff from different units and disciplines to voice concerns and develop a shared vision in a safe environment. The next step is the creation of implementation strategies that can address these challenges and enhance the development and delivery of PCCM in LMICs.

## Supplementary Material

Table S1 – Actionable ideas developed by participants on the final day of the workshop to promote collaboration or mitigate deleterious feedback loops that undermined productivity.

This is a list of supplementary files associated with this preprint. Click to download.

• SupplementalTable1PediatricCriticalCareImplementationinResourceLimitedSettingsVersion4.docx

## Figures and Tables

**Figure 1-:**
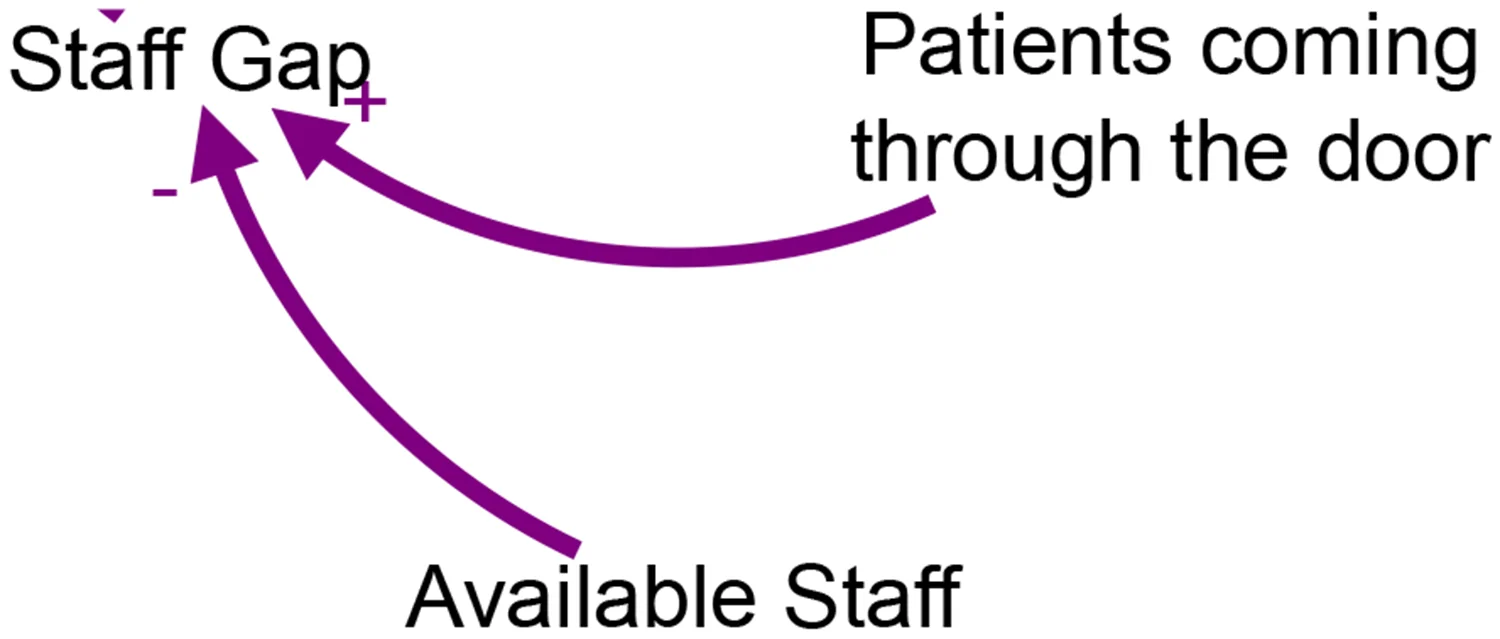
Diagram showing a positive and negative causal link

**Figure 2-:**
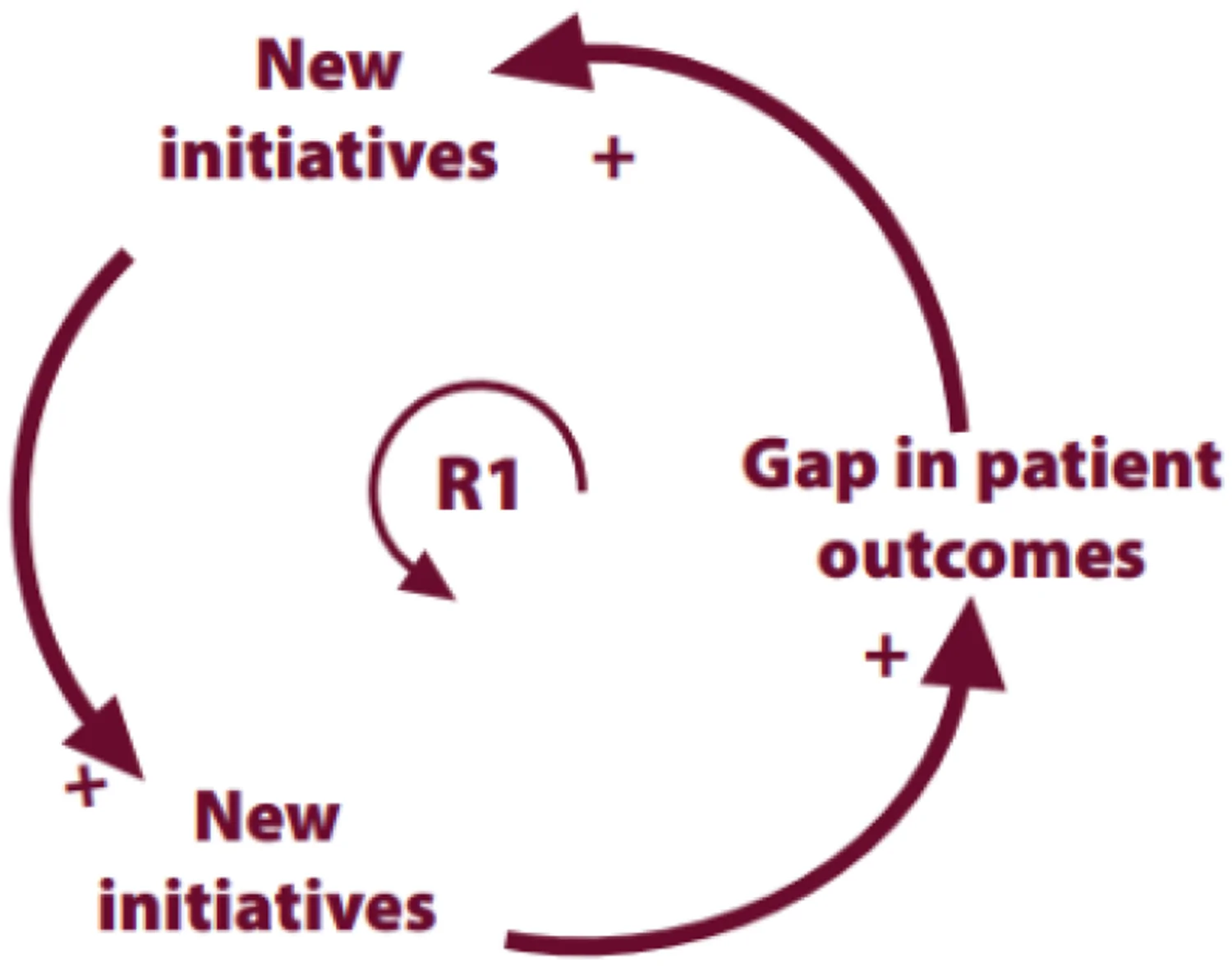
Diagram showing reinforcing loop

**Figure 3-:**
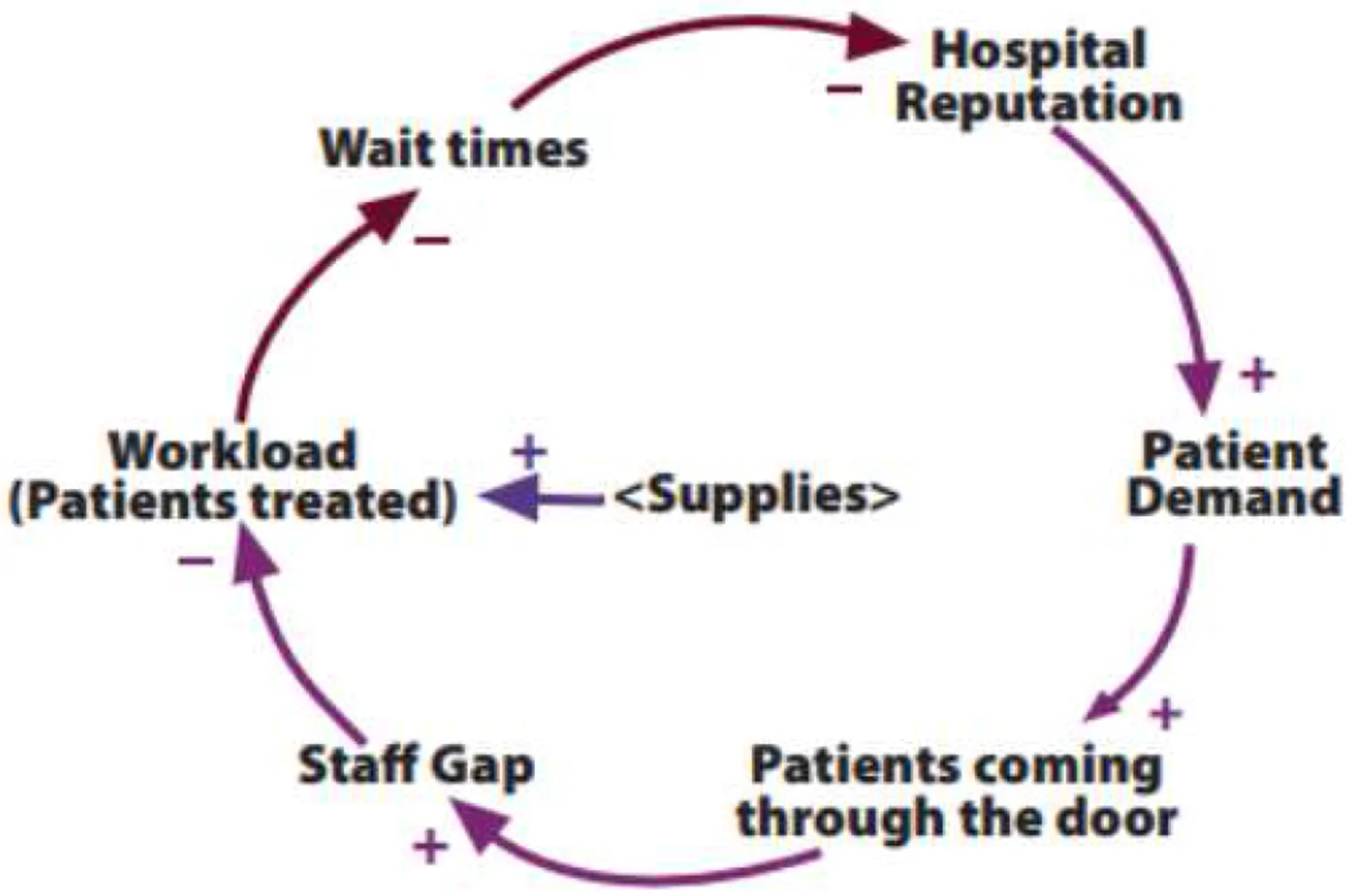
Diagram showing the balancing loop

**Figure 4-:**
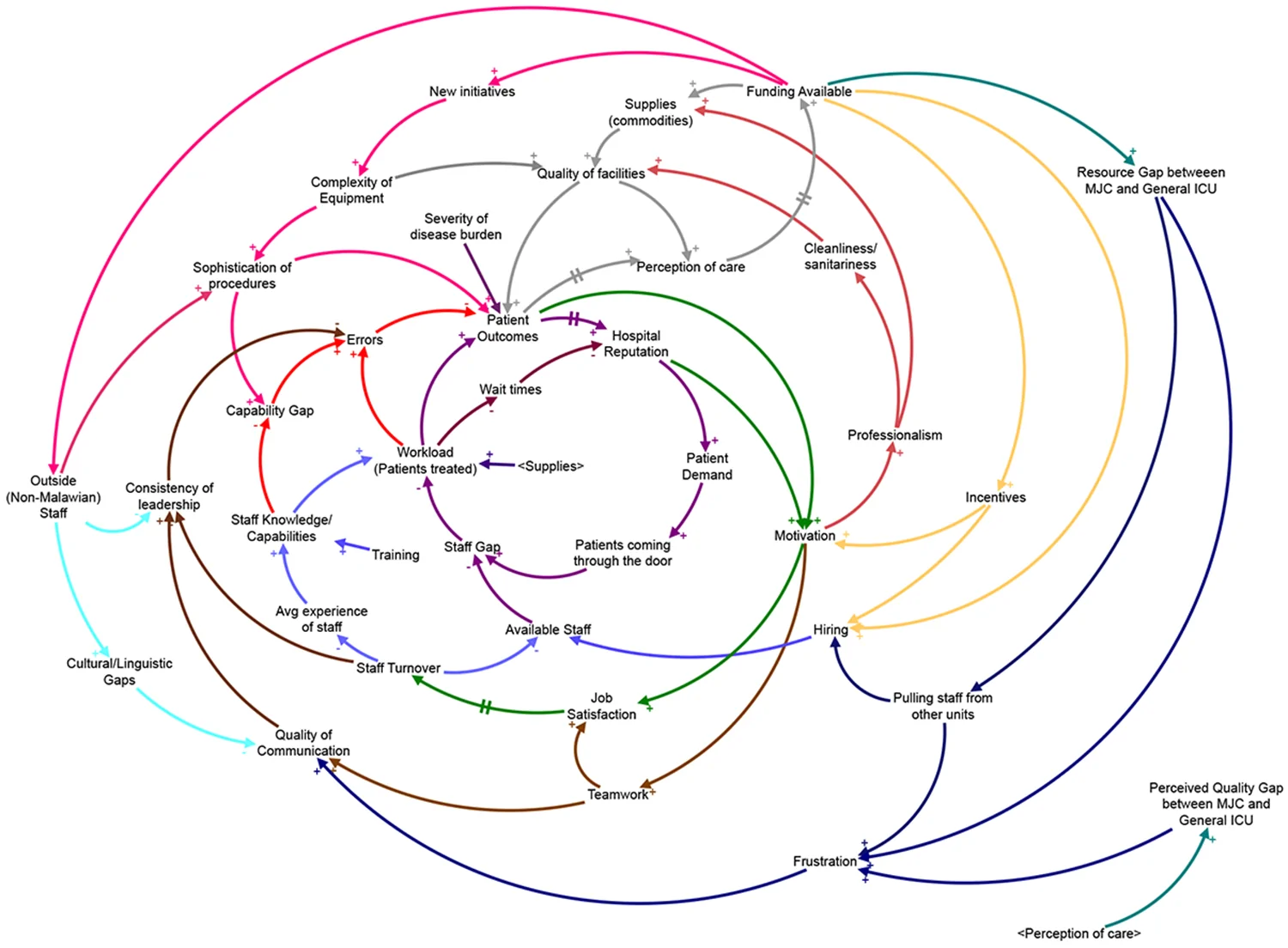
Diagram showing a synthesized causal map, which was compromised by the individual's participatory groups' causal maps

**Table 1-:** Description of loops in synthesized causal map

Colour of loop in causal map diagram	Description of relationship of variables in causal map diagram
Barrier 1: Properly trained and compensated *staff*	
Light Blue, Green and Purple Loops	Increasing motivation and hiring leads to increased available staff and decreased staff turnover, which will increase the average experience of staff and thus staff knowledge and capabilities, which improves workload and leads to improved patient outcome
Purple and Dark Red Loops	When the number of patients coming through the door increases, the staffing gap increases, leading to an increased workload for staff, creating more room for errors and lowering the quality of care. This eventually leads to a decline in the hospital's reputation in Malawi, decreasing the number of patients coming through the door.
Brown and Light Red Loops	Lack of leadership consistency leads to increased errors, negatively impacting patient outcomes. Additionally, errors increase because of increased workload and the gap between necessary staff knowledge and capabilities and actual.
Baby Blue and Light Red Loops	With the increase in staff and visitors coming from outside Malawi, the cultural/ linguistic gap increases, reducing the quality of communication between the staff.
Barrier 2 & 3: Equipment and Space	
Yellow Loops	As funding increases, incentives increase, which leads to increased motivation and hiring. Increasing funding can also lead to increased hiring by itself.
Pink Loops	As new initiatives increase, the sophistication of medical procedures increases, too. This results in a higher target for staff capabilities and thus increases the capability gap.
Gray	Funding increases commodities, improving the quality of facilities and patient outcomes, leading to improved care and more funding availability.
Barrier 4: System	
Dark Blue	Increased funding into PICU leads to a resource gap between low-resource and high-resource units. Additionally, the new funding leads to increased hiring in high-resource units, which leads to staff pulling from other hospital units. Both things lead to increased frustration within the staff of low-resource units and decreased communication quality.

## Key Points:

1. Given the significant burden of critical illness and high mortality in LMICs, ICU care could substantially improve patient outcomes in LMICs
2. To evaluate the actual and perceived barriers and facilitators of establishing pediatric ICUs in LMICs, we employed system dynamics, an approach designed to understand the behavior of dynamically complex systems
3. Group model building process allows understanding of barriers and facilitator in implementing, functioning, and sustainability of a PICU in lower resource settings.
4. GMB allows the development of a system-shared mental model among the mutliple stakeholders
